# Government infrastructure investment stimulation through booming natural resources: Evidence from a lower-middle-income country

**DOI:** 10.1371/journal.pone.0301710

**Published:** 2024-05-16

**Authors:** Bachtari Alam Hidayat, Yesi Hendriani Supartoyo, Sigit Setiawan, Ragimun Ragimun, Zamroni Salim

**Affiliations:** 1 The National Research and Innovation Agency, Jakarta, Republic of Indonesia; 2 Research Center for Macroeconomics and Finance, The National Research and Innovation Agency, Jakarta, Republic of Indonesia; University of Ghana, GHANA

## Abstract

The dynamics of central government funding to regions depend on local investments. In regional autonomy, local governments are encouraged to be more self-reliant from the central government. For regions with high natural resource yields, they will not encounter difficulties in meeting their fiscal needs. Community welfare can be realized through fulfilling basic needs, one of which is infrastructure development. High-quality infrastructure will be able to contribute to further progress in trade, thus enhancing production efficiency. The objective of this research is to analyze the extent of the influence of central government transfer funds, especially the Natural Resource Revenue Sharing Funds (DBH SDA), on local government investments in infrastructure across 508 districts/cities in Indonesia. The method used is dynamic panel regression using the Generalized Method of Moment (GMM) Arellano-Bond approach. This study finds that the role of DBH SDA is still low in infrastructure spending. The role of the central government remains significant in determining infrastructure spending at the district/city level in Indonesia. This indicates that local governments rely more on other sectors in infrastructure investment. By enhancing the role of DBH SDA through technological advancements, it is hoped that the market value of natural resources can be higher through resource downstreaming. This strategy will have broader impacts, as labor needs can be absorbed not only in raw material production activities but also in the processing technology sector. Furthermore, the utilization of natural resources with modern technology can increase extraction efficiency, support sustainable development, and minimize environmental impacts.

## Introduction

Indonesia has been implementing regional autonomy since 2001, with the hope of improving the effectiveness and results of government administration in the regions, especially in the implementation of development and services to the community, as well as to improve the development of political stability and national unity. In principle, regional autonomy gives regions the right to manage their governance through independent decision-making in the areas of social, economic, and infrastructure development. With regional autonomy, each local government should already have sufficient capabilities to lead and manage every aspect of government and development. Autonomy allows regions to initiate development with available resources. For regional development, budget planning is needed, the amount of which is adjusted to the financial capacity of the region, including the revenue generated by the region. This requires regions to have sources of income in various ways.

In driving the economy, local governments play a role in issuing expenditures for stimulus and productive public needs. The source of local government spending is generated from local revenues, be it local own-source revenues, balancing funds, and other legal local revenues. A region in the era of autonomy can be independent if local revenues can play a major role in regional spending. However, not all regions have adequate resources and management to increase the role of local revenues in financing local expenditures.

Fiscal balance transfers are an indicator of the fiscal decentralization that the central government has imposed on local governments [1]. The State Budget (APBN) is one of the sources of regional income, which is channeled through balancing funds. These balancing funds consist of taxes and natural resources, general allocation funds, and special allocation funds, which aim to balance the fiscal balance of the central and regional governments, and improve public services and community welfare. These objectives are often difficult to achieve, due to regional financial management policies that focus more on personnel expenditure.

Regions with abundant natural resources, but without the ability to manage the benefits of natural resources, will lose the opportunity to improve welfare and economic growth. This inadequate way of managing budgets derived from natural resources can also be exacerbated if local governments do not pay attention to sustainability and efficiency factors. This is a challenge for the regions concerned. In comparison, natural resource management policies in countries in the Scandinavian region have undergone changes that lead to sustainability through the use of technology, innovation, and strong institutions [2].

In addition, several studies also explain that the trend of consumption of natural resources, such as mining, will continue to increase [3]. Many countries have successfully utilized their natural resources as the main economic driver [4–6]. Many developed countries have focused on natural resources as the main source of income [7, 8]. A country’s wealth and global economic status can be reflected in its natural resources. Sustainable natural resource utilization policies can be structured based on the value of these resources, with each country having economic growth priorities in line with the scale of its natural resources [9, 10]. Therefore, natural resource management must be done carefully and exploratively. A country’s development index is often described through the work of its people related to the utilization of natural resources [11, 12]. Currently, the direction of world development is no longer only focused on the welfare of the present, but must also ensure a better situation in the future [13–16]. Sustainable development is the basic guideline for the direction of global development, which pays more attention to social, economic and environmental life in a balanced manner [17, 18].

The policy of regional autonomy was born with the assumption that local governments and their communities are the ones who know the needs and appropriate service standards. In addition, local governments are considered to have adequate knowledge of the development approach that best suits the needs of their communities. The central government in the regional autonomy scheme is a supporter of development through transfer funds, and in the end, policy execution and implementation are carried out directly by local governments. With regions having more independent authority in development, compared to a centralized regime, it can accelerate equitable development and economic improvement.

The objective of the balancing funds (Revenue Sharing Fund, Special Allocation Fund, General Allocation Fund) is to achieve equality and fairness between the central government and local governments in development and public services. Balancing funds are designed to address fiscal imbalances, ensuring that regions with lower economic potential can still provide quality public services. In addition, the purpose of the balancing fund is to provide financial support to regions that have limited resources to improve the welfare of their communities. As such, the balancing fund is an important instrument in maintaining stability and equitable development across the region.

Several literatures highlight natural resource management and infrastructure investment. They support the idea that investment in infrastructure and wise natural resource management can contribute to sustainable economic growth and improved social conditions. Some studies explain that economic growth can be triggered through infrastructure investment. That is, increasing infrastructure development capital contributes to economic growth [13, 19, 20]. Countries can escape the resource curse if they use revenues from natural resources for public investment [21]. Natural resources are more likely to have an impact on capital formation through public spending [22–25]. Infrastructure investment policies may have a greater marginal impact when implemented in lagging regions. Infrastructure investment can have a positive impact on economic growth through easier trade [26, 27].

Under proper management, natural resource wealth will be linear with the welfare of a country. With efficient management and synergy with technology, natural resources can have added value, and have less vulnerability to market price shocks, than countries that only focus on exporting raw materials [28–33]. But when there is no technological innovation to create added value, it is necessary to have an appropriate strategy for optimal utilization of natural resource benefits, which involves strong and committed institutions to support sustainable development [34]. One of the ways to utilize natural resource returns is through infrastructure investment. Infrastructure is important for economic growth because it provides a foundation for economic activity, increases productivity, facilitates mobility, and attracts investment, creating a conducive business environment. Infrastructure is an important investment target in utilizing natural resource benefits with limited wealth distribution between regions [35]. However, a common problem is that investment in this sector is still low, especially in countries rich in natural resources [36], so that economic growth is not truly driven by the potential of natural resources [37]. Adequate infrastructure will remove barriers to sustainable development and contribute to the achievement of several SDGs [38, 39]. There is causality between infrastructure and sustainable economic growth and investment in infrastructure has a positive effect on sustainable economic growth rates [40].

Indonesia is known as a country that is rich and has tremendous diversity in natural resources. Indonesia’s natural wealth includes mineral mines, biodiversity, and fertile agricultural land. After exercising regional autonomy since the early 2000s, regional independence in Indonesia should be achieved to meet the basic needs of its people. Regional income from natural resources can be utilized to accelerate economic growth through the provision of adequate and equitable infrastructure facilities. Research on economic development can be driven by the wealth of natural resources available [41–43]. However, regions with abundant natural resources also have the potential for social conflict [44–46]. The challenge facing regions with abundant natural resources is to utilize them for the benefit of the wider community. The purpose of this study is to investigate the extent of the influence of central government transfer funds, especially Natural Resource Revenue Sharing Funds (DBH SDA), on infrastructure investment in local governments in Indonesia. We also discuss natural resources, albeit briefly addressing their efficient use, sustainable development. However, we limit the discussion regarding environmental impacts, climate change, or the climate crisis.

## Method

This type of research is quantitative research. The secondary data collection is sourced from the financial records of the Ministry of Finance and the Central Statistics Agency from the year 2010 to 2020. The research sample is 508 districts and cities in Indonesia. The sampling technique used was total sampling. The year 2010 marked an important momentum for Indonesia as it experienced a resource boom. This means that during this period, Indonesia saw an increase in global demand for natural resource commodities, accompanied by rising commodity prices, resulting in a positive impact on revenue generation for countries or regions abundant in natural resources during the era of regional autonomy and fiscal decentralization. Therefore, the research period begins in 2010.

Dynamic panel regression method with Generalized Method of Moment (GMM) Arellano-Bond approach is used to test this research. Generalized Method of Moments (GMM) is a statistical method used to estimate parameters in econometric models, minimizing the difference between theoretical and observed values. The advantages of Generalized Method of Moments (GMM) include its ability to handle heteroscedasticity and serial correlation problems, provide flexibility in instrument selection, and efficiency in dealing with nonlinear models [47].

The model in this study refers to infrastructure per capita (INFRAKap) as the dependent variable, which is determined by Natural Resources Revenue Sharing Fund per capita (DBH SDAKap), General Allocation Fund per capita (DAUKap), Regional Original Revenue per capita (PADKap), Special Allocation Fund per capita (DAKKap) and Income per capita (PDRBKap). The formulation in the logarithmic function is as follows:

Log INFRAKap _it_ = γ_0_ + γ_1_ Log DBH_SDAKap_it_ + γ_2_ Log DAUKap_it_ + γ_3_ Log DAKKap_it_ + γ_4_ Log PADKap_it_ +γ_5_ Log PDRBKap_it_ +ɛ_it_

Where

LogINFRAKap = Log Government expenditure on district infrastructure

LogDBH SDAKap = Log Natural Resource Revenue Sharing per capita

LogDAUKap = Log General Allocation Fund per capita

LogDAKKap = Log Special Allocation Fund per capita

LogPADKap = Log Local Own-Source Revenue per capita

LogPDRBKap = Log Gross Domestic Regional Product/GDRP per capita

γ_0_ = Constant

γ_1_… γ_5_ = Paramater Value of Variables

i = Regency / City

t = 2010–2020

ε1 = Error term

This model was established to measure the simultaneous influence of the variables of APBD revenue components (DBH_SDA, DAU, DAK, PAD) and per capita income (PDRBkap) on government expenditure allocated to infrastructure (INFRAKap). Data analysis in this study was conducted with an econometric model using panel data analysis.

## Result

Indonesia has regencies and cities spread across many islands. The following shows the average infrastructure investment in the six major islands of Indonesia.

**Fig 1:**
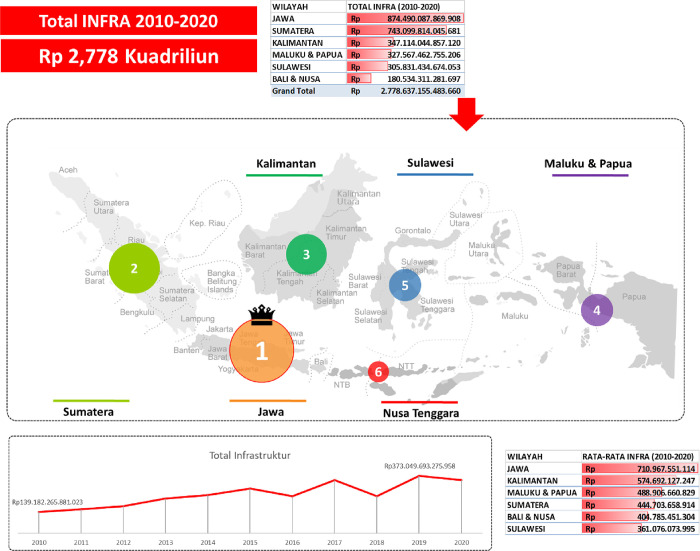
Total and average infrastructure expenditure values by region in Indonesia, 2010–2020. Source: Compiled from Regional Financial Statistics, Ministry of Finance, Republic of Indonesia.

During the period 2010 to 2020, in Fig 1, the total infrastructure spending (INFRAKap) received reached IDR 2.778 quadrillion. Java is the region with the highest INFRAKap value, followed by Sumatra, Kalimantan, Maluku & Papua, Sulawesi, and finally Bali & Nusa Tenggara. The average value of INFRA during the same period was also owned by the Java region, then Kalimantan, Maluku & Papua, Sumatra, Bali & Nusa Tenggara, and finally Sulawesi. The highest total INFRA value occurred in 2019, and 2010 was the lowest total value during this period.

**Fig 2:**
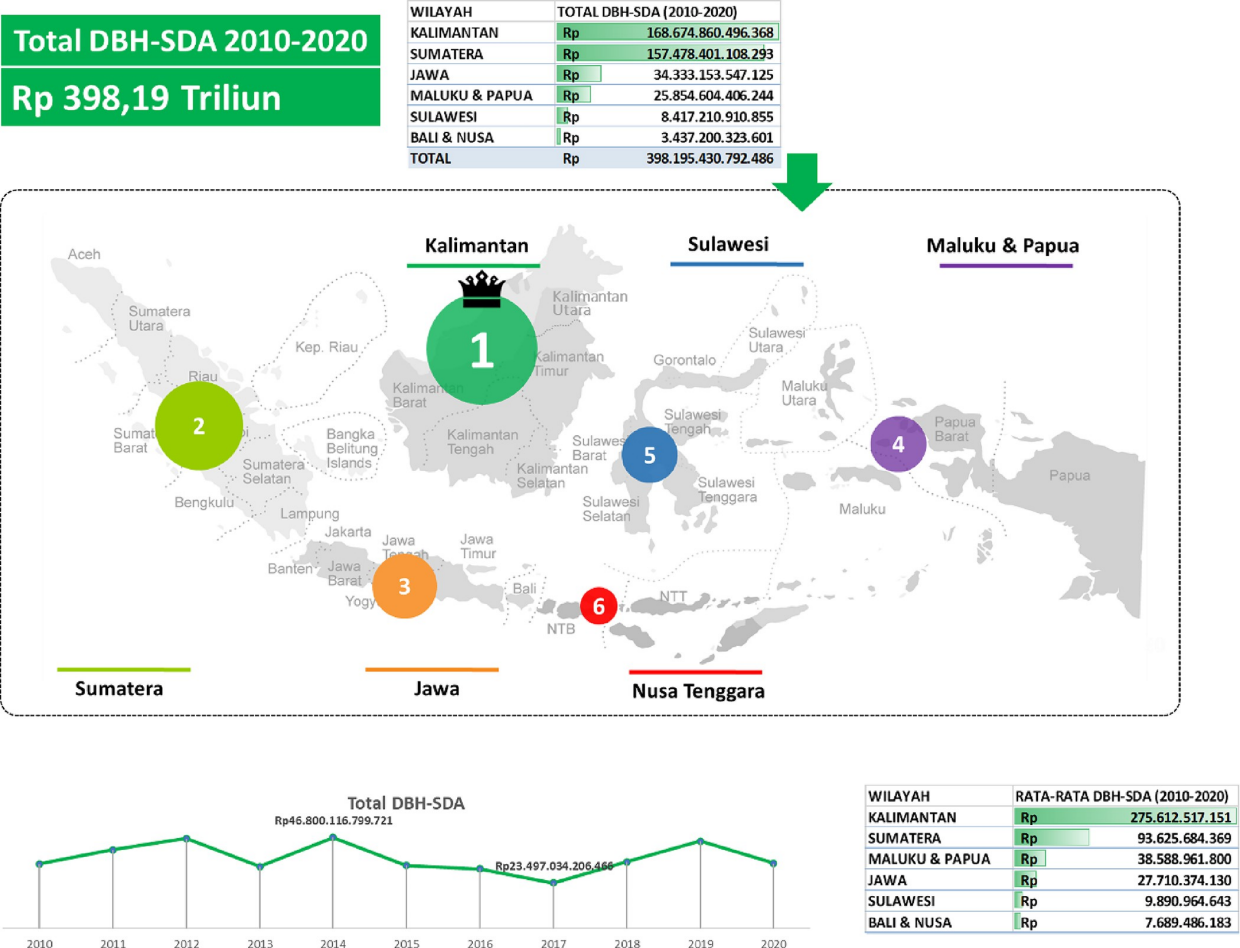
Total and average natural resource revenue sharing funds (DBH-SDA) by region in Indonesia, 2010–2020. Source: Compiled from Regional Financial Statistics, Ministry of Finance, Republic of Indonesia.

During the period of 2010 to 2020, in Fig 2, the total Natural Resource Revenue Sharing Funds (DBH-SDA) received amounted to Rp 398.19 trillion. Kalimantan emerged as the region with the highest DBH-SDA value, followed by Sumatra, Java, Maluku & Papua, Sulawesi, and lastly Bali & Nusa Tenggara. The average DBH-SDA value during the same period was also dominated by Kalimantan, followed by Sumatra, Maluku & Papua, Java, Sulawesi, and finally Bali & Nusa Tenggara. The highest total DBH-SDA value occurred in 2014, while the lowest total value was recorded in 2017 during this period.

### Generalized Method of Moment (GMM) equation

The method used in this study is dynamic panel data regression using the GMM Arellano-Bond approach. The following are the results of panel regression calculations using the help of the STATA 17 program.

The results of the GMM, estimation calculation in Table 1 are explained by the following equation:

**Log InfraKap = -9**.**72 + 0.01 LogDBH SDAKap + 0.17 LogDAUKap—0.07 LogDAKKap + 0.26 LogPADKap + 1.27 LogPDRBKap**

The above equation can be interpreted as follows:

1. γ0 is -9.72 which means that if DBH SDAKap, DAUKap, DAKKap, PADKap and PDRBKap are zero percent, then ZINFRAKap will be -9.72 percent.
2. The coefficient of DBH SDAKap is 0.01, which means that if there is an increase in DBH SDAKap by 1 percent (assuming other variables are constant), then ZINFRAKap will increase by 0.01 percent.
3. The DAUKap coefficient is 0.17, which means that if there is an increase in DAUKap by 1 percent (assuming other variables are constant), then ZINFRAKap will increase by 0.17 percent.
4. The DAKKap coefficient is -0.07, which means that if there is an increase in DAKKap by 1 percent (assuming other variables are constant), then ZINFRAKap will decrease by 0.07 percent.
5. The PADKap coefficient is 0.27, which means that if there is an increase in PADKap by 1 percent (assuming other variables are constant), then ZINFRAKap will increase by 0.27 percent.
6. The PDRBKap coefficient is 1.27, which means that if there is an increase in PDRBKap by 1 percent (assuming other variables are constant), then ZINFRAKap will increase by 1.27 percent.

**Table 1:** Model estimation results and partial effect of infrastructure expenditure model per capita.

Log_INFRAKap	Coefficient	Robust	z	P>|z|	[95% conf.	interval]
		std. err.				
Log_INFRAKap						
L1.	-0.2	0.02	-11.08	0. 00	-0.24	-0.17
Log_DBH_SDAKap	0.01	0.02	0.56	0.58	-0.03	0.05
Log_DAUKap	0.17	0.07	2.48	0.01	0.37	0.31
Log_DAKKap	-0.07	0.02	-3.03	0. 00	0.11	-0.02
Log_PADKap	0.26	0.04	7.48	0. 00	0.19	0.33
Log_PDRBKap	1.27	0.19	6.81	0. 00	0.91	1.64
_cons	-9.72	2.54	-3.83	0. 00	-14.7	-4.74

Source: Processed secondary data, 2023

### Statistical hypothesis testing

#### F-test

Based on the model testing that has been done, the following are the simultaneous test results using GMM:

Based on Table 2, it is obtained that the prob value (Wald chi2) is 0.000000 <0.05; then H0 is rejected, which means that DBH_SDAKap, DAUKap, DAKKap, PADKap and PDRBKap together are able to explain ZINFRAKap significantly or in other words the model formed is fit.

**Table 2:** Results of simultaneous effect of infrastructure expenditure model per capita.

Arellano–Bond dynamic panel-data estimation	Number of obs	=	3,698
Group variable: kab_kota		=	493
Time variable: Tahun			
	Obs per group:		
	min	=	1
	avg	=	7.501014
	max	=	8
Number of instruments = 42	Wald chi2(6)	=	1205.41
	Prob > chi2	=	0

Source: Processed secondary data, 2023

#### Convergence speed calculation results

Dynamic panel estimation is useful because it includes lag variables of the dependent, allowing analysis of adjustment dynamics, such as convergence to the same value across city districts. The speed of convergence of per capita infrastructure spending is also evaluated for each region.

Based on Table 3, it is known that the convergence speed of infrastructure spending per capita between city districts is 158.99%, meaning that every year the convergence or infrastructure spending per capita between city districts decreases by around 158.99%.

**Table 3:** Calculation results of speed of convergence.

Estimated value with first difference GMM	Speed of Convergence
-0.2039	1.5899

Source: Processed secondary data, 2023

#### Short-term and long-term effects

Through the first difference GMM method, in addition to detecting the speed of convergence, it is also possible to identify the short-term and long-term effects of the independent variables on the dependent variable. The following are the results of the analysis.

Based on Table 4, it can be seen that in the short term DBH SDAKap affects INFRAKap by 1.01%, DAUKap affects INFRAKap by 17.29%, DAKKap affects INFRAKap by -6.68%, PADKap affects INFRAKap by 26.29%, and PDRBKap affects INFRAKap by 127.49%.

**Table 4:** Short-term and long-term effects.

Independent Variabel	Short-Term	Long-Term
DBH SDAKap	0.0101	-0.0311
DAUKap	0.1729	0.2518
DAKKap	-0.0668	-0.0213
PADKap	0.2629	0.2005
PDRBKap	1.2749	0.7222

Source: Processed secondary data, 2023

In the long term, DBH SDAKap affects INFRAKap by -3.11%, DAUKap affects INFRAKap by 25.18%, DAKKap affects INFRAKap by -2.13%, PADKap affects INFRAKap by 20.05%, and PDRBKap affects INFRAKap by 72.22%. Table 4 shows that DBH-SDAKap has a long-term decline.

The five independent variables used (DBH SDAKap, DAUKap, DAKKap, PADKap, and PDRBKap) have a significant influence on government spending on infrastructure. Partially, the influence of DBH SDAKap proved to be insignificant in influencing government spending on infrastructure when compared to DAUKap, PADKap, and PDRBKap. The PDRBKap variable is the independent variable that has the greatest influence where every change of 1 unit increases infrastructure investment by 1.2749 (assuming other variables are constant). In order of the largest regression coefficient value in influencing local government spending in infrastructure are 1) PDRBKap, 2) PADKap, 3) DAUKap, 4) DBH SDAKap. Meanwhile, DAKKap has a negative influence on increasing infrastructure investment.

## Discussion

Infrastructure investment in Indonesia during 2010–2020 has increased, when compared to 2010, the total value of infrastructure investment in 2020 has increased by 147.79%, while for the same period, DBH SDAKap has increased by 462.91% (Figs 1 and 2). Given that the vision and mission of the Indonesian government for the 2014–2019 period as stated in the National Medium-Term Development Plan (RPJMN) 2015–2019, infrastructure development has an important role in the national development strategy. This is in line with the theme of the 2016 government work plan (RKP), namely Accelerating Infrastructure Development to Strengthen the Foundation for Quality Development; the infrastructure budget in the state budget can be classified into 3 major groups, namely economic infrastructure, social infrastructure, and infrastructure support. Economic infrastructure is intended for the development (including maintenance) of facilities and infrastructure needed for the smooth mobility of the flow of goods and services, as well as the smooth production process. Therefore, the government must utilize revenues from the natural resource sector to increase investment in infrastructure. The government will make development expenditures as a step to carry out these functions. Development spending is government spending to meet development needs. One of the objectives of development is to improve people’s welfare. Community welfare can be realized by fulfilling basic needs such as the availability of inclusive infrastructure.

From the analysis results, it is evident that infrastructure spending in Indonesia is more influenced by Regional Retribution (PDRB Kap) and Regional Original Income (PAD Kap) compared to Natural Resource Revenue Sharing Funds (DBH SDA Kap) (Table 1), which means that the role of the central government is still so large in determining infrastructure spending at the Regency/City level in Indonesia. Meanwhile, with the exploitation of natural resources, infrastructure development should occur. Resource exploitation does not have significant spillover effects on some sustainable contributors such as infrastructure, especially in developing countries [48]. Natural resources should not be the target of criticism for their suboptimal role, instead focus should be placed on policies and measures that can improve natural resource management [49].

Compared to Natural Resource Revenue Sharing Funds (DBH-SDA Kap), it turns out that Regional Retribution (PDRB Kap) plays a dominant role in infrastructure investment in Indonesia. The higher the Regional Gross Domestic Product (PDRB) of an area, the higher the economic growth, leading to an increase in regional expenditure allocation for public services. Therefore, under normal circumstances, an increase in PDRB leads to greater government revenue, resulting in increased government expenditure as well. This is in line with research [28] which generally shows that gross regional domestic product has a positive effect on regional spending. This is in accordance with the theory which states that if PDRB Kap increases, the income received by the factors of production owned by various groups of people will also increase, so that from this income the community will buy goods and services for both consumption and investment purposes. The higher the PDRB of a region means that economic growth is increasing, which results in an increase in the allocation of regional expenditure for public services. Therefore, under normal circumstances, the increase in PDRB causes greater government revenue, as well as greater government spending. Development expenditure has a positive impact on Gross Domestic Product (GDP) growth, which suggests that investment in development can support economic growth [28, 50–52]. Furthermore, results show that investing in infrastructure can accelerate economic growth. Furthermore, results show that both education and life expectancy at birth have a positive impact on GDP growth. Thus, increased infrastructure investment in education and health facilities can accelerate economic growth [53, 54].

Observing that the role of DBH SDA Kap is still below that of other income sources, the policy of the Indonesian government is to increase the market value of Indonesia’s natural resources. Enhancing the processing technology of natural resources to increase their market value through downstreaming is one approach. The policy of natural resource downstreaming will be implemented to enhance the role of DBH SDA. This strategy will have broader impacts, as labor needs can be absorbed not only in raw natural resource processing but also in the technology processing sector. Additionally, utilizing natural resources with modern technology can increase extraction efficiency, support sustainable development, and minimize environmental impacts.

Downstreaming of resources, through advanced processing, can increase the added value of these natural resources. For example, in the mining industry, raw mineral resources can be processed into finished products with higher value, such as metals or chemicals. Downstreaming of natural resources allows for economic diversification in the region. For instance, by utilizing modern technology in wood processing, a region that previously relied on exporting raw timber can switch to producing furniture or wooden building materials. Expanding the processing sector with advanced technology can create new job opportunities. This not only includes direct employment in the processing process but also related jobs such as software development, machine maintenance, and operational management.

Modern technology enables more efficient extraction and processing of natural resources, reducing waste and energy consumption in the process. This supports the principle of sustainable development by prolonging the lifespan of resources and reducing environmental impacts. Investment in processing technology also means investment in research and development (R&D) to create innovative solutions. DBH SDA can be used to support R&D programs focused on developing more environmentally friendly and efficient processing technologies.

By implementing the latest technology in natural resource processing, a country or region can enhance its competitiveness in the global market. Products produced with higher quality and lower production costs will be more competitive in the international market. In implementing the natural resource downstreaming strategy, it is important to consider partnership opportunities between the private sector, government, and research institutions. This can help accelerate technology transfer and ensure sustainable investment in infrastructure and human capacity development. Considering all of these factors, the role of DBH SDA in supporting natural resource downstreaming efforts becomes crucial. Proper funding can be provided for projects that have the potential to increase added value, efficiency, and sustainability in natural resource processing.

Thus, for local governments that are endowed with relatively larger natural resources, the Revenue Sharing Fund received will be able to meet their fiscal needs. Meanwhile, local governments that lack natural resources can explore existing taxation potential. If the Revenue Sharing Fund received is inadequate, the central government provides the General Allocation Fund (DAU) and the Special Allocation Fund (DAK) as a regional financial balance in order to carry out the duties and functions of government and public services. Revenue from resources obtained, the government needs to decide how much resources to take in the current period and how much to leave for the future [41].

Similar to fiscal decentralization in China, fiscal decentralization in India is believed to facilitate the provision of local public goods that enhance social infrastructure and support poverty alleviation policies [55]. However, its impact on economic growth remains debated. Studies conducted by [56] highlight the significant impact of revenue decentralization on infrastructure investment at the regional level. Their findings indicate that revenue decentralization in 20 European countries during the period 1990–2009 led to a significant increase in expenditure on productive infrastructure at the local level. Furthermore, this research emphasizes that revenue decentralization provides incentives for local governments to allocate more resources to infrastructure policies that promote economic growth rather than redistribution purposes. This shift in focus is considered crucial for long-term growth and economic development. Therefore, revenue decentralization plays a crucial role in promoting regional infrastructure investment, which is not only important for economic growth but also for improving public services and overall local development.

Revenue from natural resources is vital to any country’s economy, and countries rich in natural resources will continue to grow more productive due to rapidly increasing demand. Natural resources have a positive impact on sustainable development, but their impact depends on the quality of governance and the level of economic development [57]. Infrastructure and natural resource extraction can have a positive impact on economic growth [58–60]. Deficiencies in transportation infrastructure tend to inhibit the benefits of other infrastructure improvements. For example, while investments in extensive and efficient telecommunications networks allow for the rapid flow of information, which increases overall economic efficiency, deficiencies in transportation infrastructure tend to hinder the gains generated by improvements in telecommunications due to the loss of reliability in business supply chains [61]. Abundance of natural resources without associated infrastructure investment will not generate prosperity in the long run. This is because the purpose of natural resource extraction is to convert inexhaustible natural resource wealth into reproducible assets with tangible rates of return such as public infrastructure, education, and foreign investment [62].

In the study by [63], natural resources play a significant role in regional development in Greece by offering comparative advantages to regions that possess them. These resources can directly contribute to the production of goods in the primary sector or attract manufacturing businesses in the secondary sector that utilize these resources. Natural resources, such as mineral resources, water resources, and resources related to tourism development, can enhance the attractiveness of regions for tourism activities, fishing, and recreation, thus shaping each region’s appeal to tourists. However, the ability of natural resources to guarantee economic development and prosperity depends on the exploitation and presence of adequate infrastructure. Regions with natural resources may not be able to exploit them effectively, leading to their absorption by regions with stronger economies. Additionally, a lack of infrastructure to support the sale of goods produced from natural resources at competitive prices can hinder economic development.

In the case of Greece, where there is strong inequality in terms of prosperity and spatial concentration, natural resources are traditionally considered important for regional economic development [63]. Despite efforts to reduce regional disparities through public investment and support for sectors such as agriculture, manufacturing, and tourism, there are still challenges in fully utilizing natural resources for sustainable regional development. Although natural resources can offer comparative advantages to a region, the exploitation of natural resources and the presence of supporting infrastructure are crucial to translating resource potential into economic development.

Good transportation infrastructure, such as roads, bridges, and airports, facilitates the flow of goods and people, improves safety and convenience, supports the distribution of goods, and enhances interregional connectivity [64–67]. This not only improves efficiency in the supply chain, but also opens up accessibility for businesses, encourages investment, and expands markets [68]. In addition, reliable energy infrastructure, such as power generation and energy distribution, supports the sustainability of economic and industrial activities [69, 70]. High-quality information and communication technology infrastructure also facilitates the rapid flow of information, encourages innovation, and strengthens the service sector [71, 72]. Public infrastructure development creates a conducive environment for economic growth by improving productivity, competitiveness, and economic resilience. It not only provides short-term benefits through job creation during development, but also provides a solid foundation for long-term development through improved operational efficiency and global competitiveness [73].

Research conducted by [74] on regional development in Eastern Macedonia and Thrace, Greece, through road transportation projects found that public expenditure on road projects correlates directly with the area of municipalities and the length of the improved road network, rather than with municipal population or Gross Domestic Product (GDP). Larger municipalities with larger land areas have higher needs for road infrastructure improvement and modernization, leading to higher allocations of public funds. For example, Soufli municipality, the largest in terms of land area, received a substantial portion of the total expenditure. This research highlights the importance of regional development policies and infrastructure investments in reducing regional disparities and promoting economic growth. Road transportation projects play a crucial role in enhancing connectivity, accessibility, and economic opportunities in Eastern Macedonia and Thrace, contributing to the overall development of the region. These findings underscore the importance of targeted infrastructure investment in advancing regional development and addressing economic challenges in specific geographic areas.

Research on infrastructure spending in Australia shows that increased government expenditure on building highways significantly reduces local unemployment rates. The study’s results indicate that areas receiving large budget allocations for highway construction experience greater reductions in unemployment rates compared to the national average [75]. However, there is a possibility that this impact could be mitigated when considering the potential responses from local governments in reducing their spending in response to increased federal funding, as well as changes in population migration patterns. Estimates of the impact on unemployment rates may not fully reflect the actual impact of infrastructure spending on the local economy. The use of federally funded infrastructure programs can stimulate local job creation, especially in the short term.

Infrastructure investment has played a significant role in supporting regional economic growth in China. Various types of infrastructure, such as electricity, highways, railways, and telecommunications, have made positive contributions to regional growth. The role of this infrastructure varies over time and across regions, especially during the period 1990–2013, which was a time of major economic reforms in China. This study used a dynamic panel data approach, treating each of the 30 provinces and municipalities in China as independent and interconnected entities. The analysis results show that overall infrastructure has a significant positive impact on China’s rapid economic growth [76].

The identification of short-term and long-term influences of DBH-SDAKap on infrastructure investment in Indonesia indicates that DBH-SDAKap has a positive impact on infrastructure investment in the short term but experiences a decrease in the long term (Table 4). This is in line with research [28] which states that initially there is a positive effect seen in the short term from natural resource rental income. However, in the long run, this effect turns negative and larger. This suggests that natural resources can provide short-term benefits but can hinder long-term economic growth. The decline in the economic contribution of natural resources in the long run can be caused by several factors. First, the exploitation potential of natural resources is limited and can be exhausted, resulting in a decline in production and income from this sector [77]. Second, depending on global demand, natural resource prices may fluctuate and decline over time [78]. In addition, the adoption of new technologies and diversification of the economy into other sectors can also reduce dependence on natural resources [78]. Selain itu, adopsi teknologi baru dan diversifikasi ekonomi ke sektor lain juga dapat mengurangi ketergantungan terhadap sumber daya alam [79]. Finally, environmental and sustainability issues may limit the sustainable exploitation of natural resources. This explains why, in the long run, the contribution of natural resources can be a potential constraint to infrastructure investment.

According to [80] in their research in Greece on rural development, it is essential to consider the environmental impacts of economic activities and natural resource use. Issues such as environmental degradation, decline in natural resource quality, and climate change can affect the sustainability of natural resource exploitation. By addressing environmental issues and sustainability, local action groups and other stakeholders can design and implement rural development initiatives that take into account long-term environmental impacts and ensure sustainable exploitation of natural resources.

Infrastructure investment is strongly associated with improved connectivity, easier access to resources and markets, increased productivity, expanded investment opportunities, and improved quality of life, which can drive improvements in a country’s economy [81]. The benefits of investing in infrastructure are not only income but also other broader benefits, such as more productive behavior, healthy behavior, and cultural behavior. The rules made by the government to encourage investment decisions in infrastructure must have a positive impact on society and all business actors that will affect the future. A good efficient use of funds from natural resource proceeds is through investment in infrastructure [2, 19].

According to [82], the key stakeholders in decision-making regarding Government Infrastructure may include various parties such as the Central Government, Local Government, Community, Private Sector, and Experts. The central government plays a crucial role in planning, managing, and overseeing government infrastructure covering various sectors such as transportation, energy, water, and others. Local governments are also key stakeholders in decision-making regarding government infrastructure at the local level, including the development of roads, bridges, and other public facilities. The community, as direct users of government infrastructure, has an interest in ensuring that the infrastructure meets necessary needs and standards. The private sector is often involved in government infrastructure projects through public-private partnerships or construction contracts, making them also significant stakeholders in decision-making. Experts and professionals in technical, financial, and legal fields also play a role in providing input and advice to decision-makers regarding government infrastructure. By engaging these various stakeholders, decision-making regarding government infrastructure is expected to be more holistic, transparent, and considerate of various relevant aspects.

According to [83], joint design principles in formulating relevant policies for government infrastructure may involve various stakeholders such as urban planners, climate change scientists, policymakers, and local communities in the process of planning government infrastructure to ensure relevant and sustainable solutions. Interdisciplinary collaboration involves various fields of knowledge such as environmental science, urban planning, and climate change science to formulate holistic and sustainable government infrastructure policies. Strong monitoring and evaluation are needed to track the effectiveness of government infrastructure policies in addressing environmental challenges and climate change and to ensure the sustainability of urban regeneration efforts. By applying these joint design principles, governments can develop more adaptive, sustainable, and responsive infrastructure policies to environmental challenges and climate change, as well as ensure the involvement of all stakeholders in the decision-making process regarding urban infrastructure.

This section explains how the flow of natural resource revenue-sharing funds has the potential to influence local government investment in infrastructure in several ways so that investment in infrastructure is a very important long-term investment [84]. Natural resources can drive investment in infrastructure [13, 20, 85]. Quality infrastructure will be key to improving welfare and development in a region. The first important channel is to improve the quality of infrastructure, not only in terms of quantity, but also quality [86].

Transportation infrastructure such as roads, ports and airports enable more efficient mobility for people and goods. Good mobility enables faster and cheaper distribution of goods, increasing productivity and economic efficiency [87–89]. Infrastructure such as electricity, clean water and telecommunications provides reliable and affordable access to essential resources. It supports various economic sectors, including industry, agriculture, and services [53, 54].

The existence of adequate infrastructure is often a determining factor for investors to invest in a region or country. Good infrastructure facilities create an attractive business environment and can trigger significant economic growth [90]. Good infrastructure, including healthcare, education, and public facilities, can improve the quality of life of the population. This can increase labor productivity and lure more skilled workers to the area [91–94].

Investment in infrastructure has great potential to improve economic equity as it affects several key aspects of the distribution of economic benefits in a country or region. First of all, adequate infrastructure creates better access to different regions, including remote or less developed areas [95]. With good transportation infrastructure, such as roads, bridges, and public transportation, people from remote areas can more easily access markets, schools, health services, and job opportunities in big cities. This reduces the access gap and allows residents in marginalized areas to take part in economic activity more effectively. In addition, adequate transportation will also have an impact on the use of buses for tourism, resulting in increased tourist visits [96].

Second, infrastructure investment can also support the growth of local economic sectors. For example, the development of electricity networks and clean water supply can encourage the growth of small and medium industries in certain areas. This creates new jobs and increases local income, which in turn can improve welfare levels and reduce economic disparities between regions. In addition, good infrastructure can also create opportunities for the development of tourism and agriculture sectors. Adequate roads and other supporting facilities can help connect tourist destinations with key markets, increasing tourism attractiveness and providing direct economic benefits to local communities [97].

Investment in infrastructure can also trigger multiplier effects [84, 98]. When the government allocates funds for infrastructure projects, it creates new demand for related goods and services, which can provide an additional boost to the local economy. For example, the construction of a highway will trigger demand for construction materials, equipment and additional labor. Thus, wise and well-targeted infrastructure investments can pave the way for more inclusive and sustainable economic growth, by reducing economic disparities between regions and strengthening the overall competitiveness of the country or region [99].

While there are many factors that influence economic growth, infrastructure is considered to be one of the most critical. In this context, it is suggested that public infrastructure development, especially in countries that have abundant natural resources, can contribute to better economic growth. While abundant natural resources can contribute significantly to economic growth, it is important to manage those resources wisely and promote infrastructure development.

## Conclusion

The abundance of natural resources can provide significant potential for regions to meet their fiscal needs. Natural resources can serve as an additional source of income, enhance local financial independence, enable infrastructure development, and contribute to improving community welfare. Therefore, wise utilization of natural resources can be a crucial factor in enhancing regional fiscal capacity and overall economic development. However, managing natural resources must be done sustainably and efficiently while considering supportive infrastructure development to maximize their benefits. There is a need to focus on policies and measures that can improve natural resource management to increase their value and also uphold environmental sustainability. Environmental and sustainability issues can limit the sustainable exploitation of natural resources, thus requiring appropriate strategies for optimal resource utilization. Abundant natural resources present opportunities for a country’s success in increasing investment in critical sectors such as infrastructure. Natural resources can provide additional income that can be allocated to infrastructure, help create efficiency in the supply chain, open investment opportunities, expand distribution networks, and markets.

## Supporting information

S1 File (ZIP)
