# Supplementary material for: Government infrastructure investment stimulation through booming natural resources: Evidence from a lower-middle-income country
Source: PLoS One. 2024 May 16;19(5):e0301710. doi: 10.1371/journal.pone.0301710 (PMC11098401; doi:10.1371/journal.pone.0301710)
Supplement: S1 File — (ZIP) [file pone.0301710.s001.zip › Data and do file/Fig 1 & 2.docx]

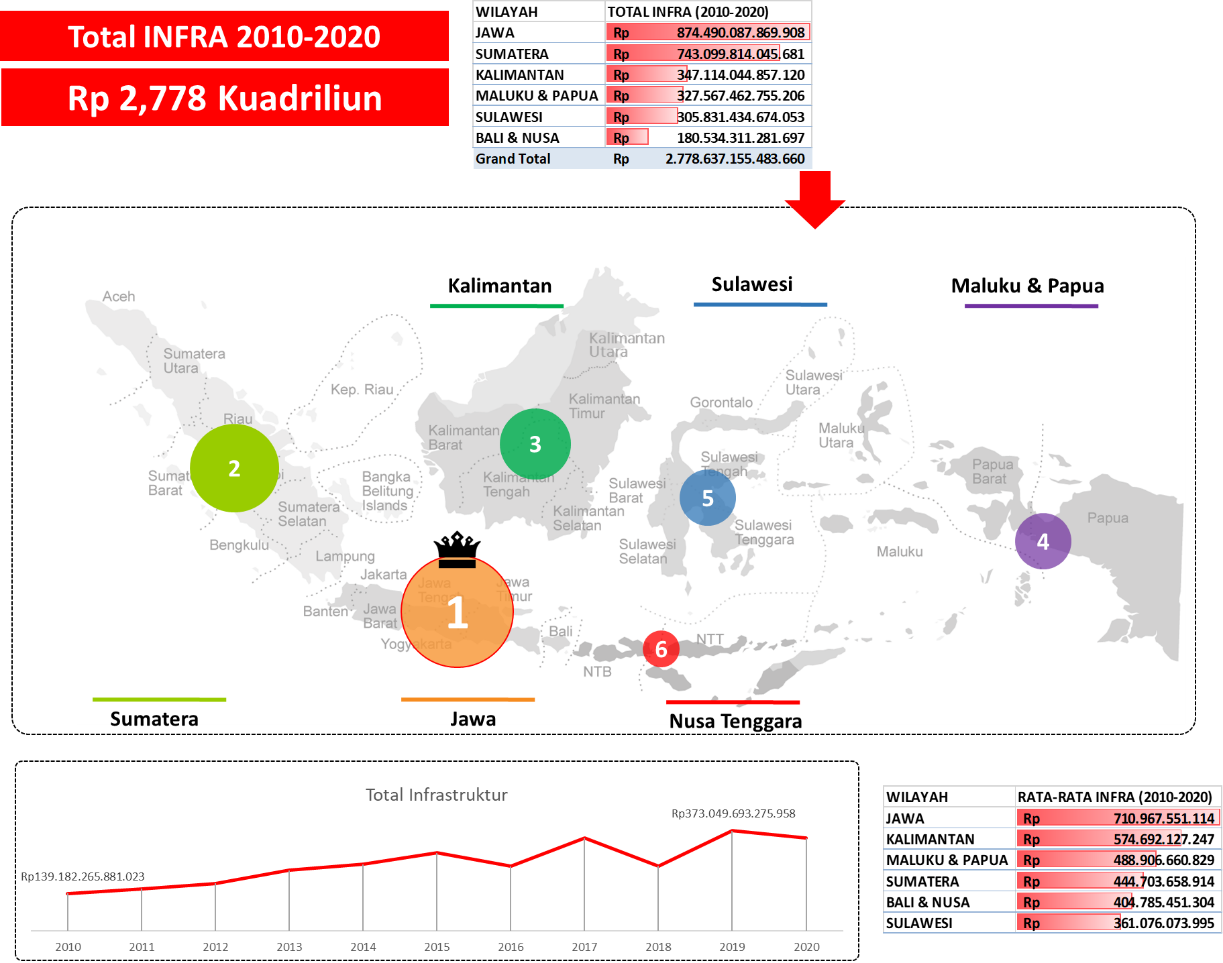


**Fig 1: Total and Average Infrastructure Expenditure Values by Region in Indonesia, 2010-2020**

Source: Compiled from Regional Financial Statistics, Ministry of Finance, Republic of Indonesia


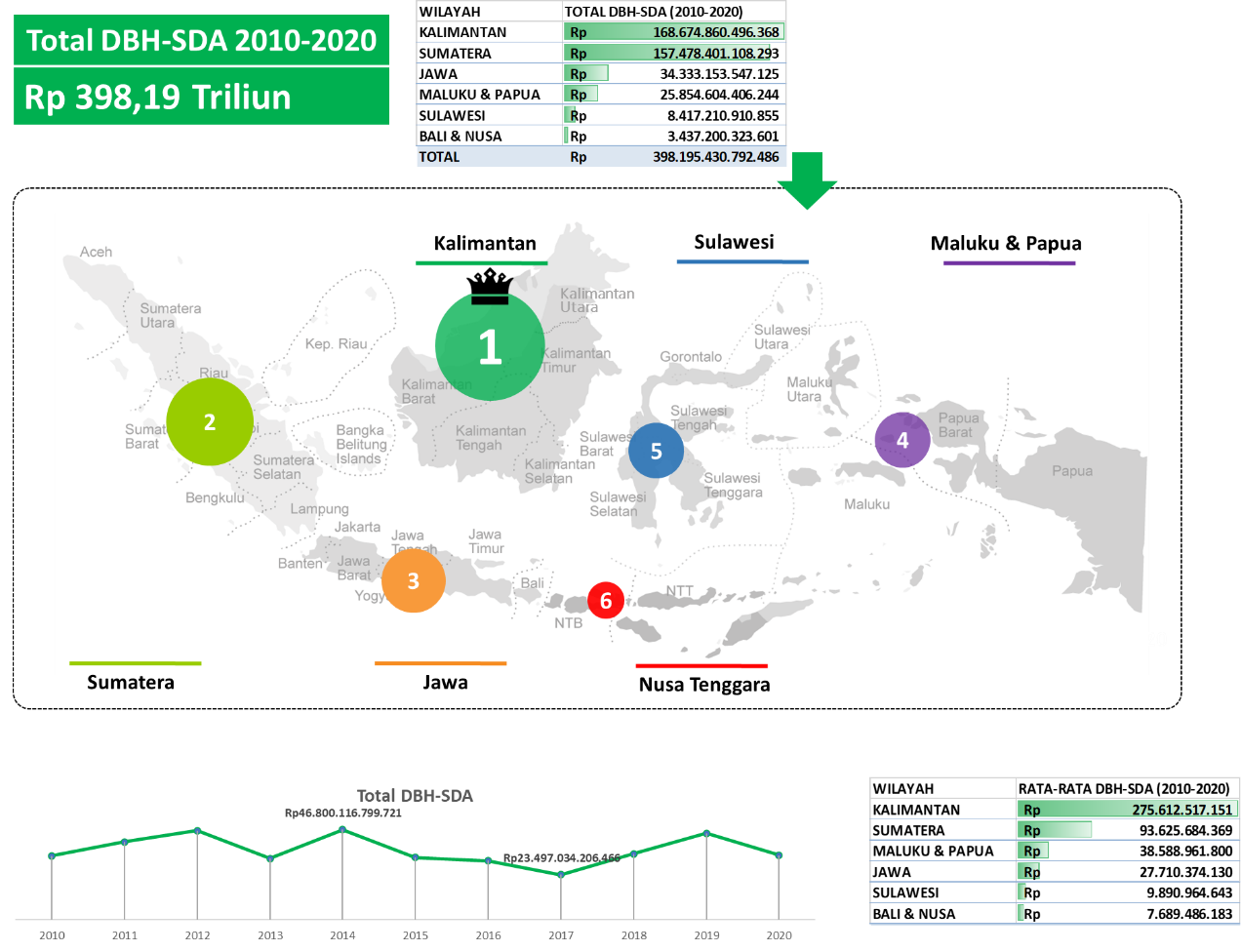


**Fig 2. Total and Average Natural Resource Revenue Sharing Funds (DBH-SDA) by Region in Indonesia, 2010-2020**

Source: Compiled from Regional Financial Statistics, Ministry of Finance, Republic of Indonesia
