# Supplementary material for: Government infrastructure investment stimulation through booming natural resources: Evidence from a lower-middle-income country
Source: PLoS One. 2024 May 16;19(5):e0301710. doi: 10.1371/journal.pone.0301710 (PMC11098401; doi:10.1371/journal.pone.0301710)
Supplement: S1 File — (ZIP) [file pone.0301710.s001.zip › Data and do file/Lampiran Output STATA - INFRA.docx]

**Lampiran Output STATA 17**

**INRASTRUKTUR MODEL**

**First Difference GMM (Table 2)**

**Robust (Table 1)**

**Kriteria GMM**

**Validitas Instrumen**

**Uji Konsistensi**

**Uji Ketidakbiasan**

**Kecepatan Konvergensi (Table 3)**

**Jangka Panjang (Table 4)**

**Jangka Pendek (Table 4)**
